# Remarks from the Editor-in-Chief

**DOI:** 10.1017/psy.2025.10039

**Published:** 2025-09-25

**Authors:** Sandip Sinharay

**Affiliations:** ETS Research Institute

Dear *Psychometrika* Readers,

Welcome to the third Psychometrika issue of 2025. Hope you enjoyed the IMPS 2025 Annual Meeting in Minneapolis. It was nice seeing many of you there in person. I encouraged an IMPS attendee to submit his paper to Psychometrika, but he said “Is it true that the review time for Psychometrika is long and authors have to make many rounds of revisions?” Probably many of you think the same way. However, I would like to inform you that the Psychometrika editorial council and the editorial team are trying really hard to ensure timely and fair reviews and publications. I very rarely choose the authors’ nightmare–“Revise and Resubmit” decision–after the 2nd round of review (and articles go to print after one or two rounds of revisions). I also take decisions on almost all revisions of conditionally accepted manuscripts by myself. We also try to send review decisions in a timely manner. Over the last one year or so, the average review time is about 2 months. Please feel free to ask some people who regularly publish or submit to Psychometrika about their recent experience with the review process.

This Psychometrika issue first includes 12 “Theory and Methods” section articles. The issue begins with three articles that focus on the analysis of ordinal data. In the first of these, Mark de Rooij, Ligaya Breemer, Dion Woestenburg, and Frank Busing present a multidimensional data analysis framework for the analysis of ordinal response variables. The second, by Bernard M. S. van Praag, J. Peter Hop, and William H. Greene, suggests a new approach for the statistical analysis of ordinal data, where the errors are supposed to be correlated. In the third article, Michael Pearce and Elena Erosheva propose a new statistical model to infer interpretable population-level preferences from ordinal comparison data. In the fourth article of this issue, Camilo A. Cárdenas-Hurtado, Irini Moustaki, Yunxiao Chen, and Giampiero Marra introduce a general framework, named Generalized Latent Variable Models for Location, Scale, and Shape parameters, for latent variable modeling. The fifth article, by Nan Zhang, Heng Xu, Manuel J. Vaulont, and Zhen Zhang, draws upon advances in machine learning including semi-supervised learning algorithms, to develop a novel method for reverse causality testing. The sixth article, by Naomichi Makino, proposes a simultaneous object and category score estimation method for joint correspondence analysis while addressing the underestimated variance problem that is inherent in correspondence analysis. In the seventh article, Jiawei Qiao, Yunxiao Chen, and Zhiliang Ying propose a constraint-based optimization method that learns an exact bi-factor loading structure from data. The eighth article, by Yongfeng Wu, Xiangyi Liao, and Qizhai Li, proposes a mathematical framework that incorporates customized factor structure as a regularization to produce the optimal orthogonal or oblique rotation in exploratory factor analysis. In the ninth article, Yon Soo Suh, Wes Bonifay, and Li Cai suggest a limited-information approach for generating item response data and apply the approach to assessing model complexity. The tenth article, by Chih-Han Leng, Ulf Böckenholt, Hsuan-Wei Lee and Grace Yao, introduces item response models for rating relational data. The eleventh article, by Naoto Yamashita, introduces a novel procedure, regression-based factor score exploration, which uniquely determines factor scores and simultaneously estimates other parameters of factor-analytic models. In the final “Theory and Methods” article to appear in this issue, Jochen Ranger, Sören Much, Niklas Neek, Augustin Mutak, and Steffi Pohl propose a series of latent trait models for the responses and the response times on low-stakes tests.

This Psychometrika issue then includes four articles from the “Application and Case Studies” section. In the first of these articles, Chengyu Cui, Yanlong Liu, and Gongjun Xu suggest new consistency results for general nonparametric classification methods for cognitive diagnosis. The second article, by Giuseppe Mignemi, Yunxiao Chen, and Irini Moustaki, introduces a latent variable model framework for analyzing peer grading data and develops a fully Bayesian procedure for statistical inference for such data. In the third article, Madeline Abbott, Walter Dempsey, Inbal Nahum-Shani, Cho Lam, David Wetter, and Jeremy Taylor suggest a continuous-time dynamic factor model for intensive longitudinal data arising from mobile health studies. In the fourth article, which is also the last article of this issue, Sun-Joo Cho, Goodwin Amanda, Jorge Salas, and Sophia Mueller incorporate a random forest (RF) approach to probe complex interactions and nonlinearity among predictors into an item response model.

Hope you enjoy the issue.

